# Childhood cancer survivorship in China: An overview of the past two decades

**DOI:** 10.1002/cam4.4831

**Published:** 2022-05-22

**Authors:** Xu Ji, Jun Su, Xinyu Liu, Ziling Mao, Wenjing Zhang, Jinhe Zhang, Xiaojie Sun, Xuesong Han

**Affiliations:** ^1^ Department of Pediatrics Emory University School of Medicine Atlanta Georgia USA; ^2^ Aflac Cancer and Blood Disorders Center Children's Healthcare of Atlanta Atlanta Georgia USA; ^3^ Center for Health Management and Policy Research, School of Public Health, Cheeloo College of Medicine Shandong University Jinan Shandong China; ^4^ NHC Key Lab of Health Economics and Policy Research (Shandong University) Jinan Shandong China; ^5^ Surveillance and Health Equity Science American Cancer Society Atlanta Georgia USA; ^6^ Department of Epidemiology, Graduate School of Public Health University of Pittsburgh Pittsburgh Pennsylvania USA

**Keywords:** childhood cancer, China, survivorship

## Abstract

Across countries in the world, China has the largest population of childhood cancer survivors. Research and care for the childhood cancer survivor population in China is fragmented. We searched studies published in English or Chinese language between January 1, 2000 and June 30, 2021, which examined various aspects of childhood cancer survivorship in China. The existing China‐focused studies were largely based on a single institution, convenient samplings with relatively small sample sizes, restricted geographic areas, cross‐sectional design, and focused on young survivors in their childhood or adolescence. These studies primarily focused on the physical late effects of cancer and its treatment, as well as the inferior psychological wellbeing among childhood cancer survivors, with few studies examining financial hardship, health promotion, and disease prevention, or healthcare delivery in survivorship. Our findings highlight the urgent need for research and evidence‐based survivorship care to serve the childhood cancer survivor population in China.

## Graphical abstract

This is the first overview of research over the past two decades that examined various aspects of childhood cancer survivorship in China, a country with the largest childhood cancer‐survivor population. We found that the existing China‐focused studies primarily focused on the physical late effects of cancer and its treatment, as well as the inferior psychological wellbeing among childhood cancer survivors, with no studies examining financial hardship, health promotion and disease prevention, or healthcare delivery during survivorship. Our findings highlight the urgent need for research and evidence‐based survivorship care to serve the growing childhood cancer‐survivor population in China.

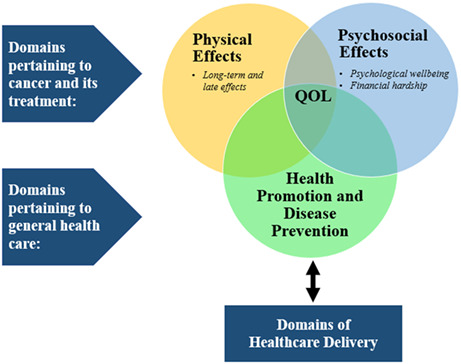

## 1 INTRODUCTION

China has the largest child population (ages 0–19 years), accounting for 13% of all children in the world.^1^ Cancer is a leading cause of death among children in China.^2^ According to the World Health Organization International Agency for Research on Cancer, 27,170 children ages 0–14 years and 9481 adolescents ages 15–19 years were diagnosed with cancer, and 10,553 children and 3574 adolescents died from cancer in China in 2020.^3^ The 5‐year prevalent cancer cases among children ages 0–14 years and adolescents ages 15–19 years in China were 92,388 and 27,640, respectively, in 2020, accounting for 14% of the prevalent childhood cancer cases worldwide.^3^ Similar to western countries, the most common cancer types among Chinese children and adolescents are leukemia, brain (central nervous system [CNS]), lymphoma, kidney, and liver cancer.^4^

The event‐free survival and overall survival of childhood cancer, especially acute lymphoblastic leukemia, have increased in China.^5^, ^6^ The growing population of childhood cancer survivors resulted from improved survival highlights, the importance of cancer survivorship research in China. Importantly, compared to the U.S. and developed countries in Europe, the survival of childhood cancer patients in China remains inferior.^7^, ^8^ For example, the overall 5‐year relative survival rate for childhood cancer was 72% in 2000–2010 in China,^4^ compared to 83% in 2003–2009 in the U.S.^9^ Treatment delay, refusal, and abandonment were found to be contributing factors to the inferior childhood cancer survival in China,^8^, ^10^ with less data on healthcare delivery and health outcomes post treatment and during survivorship. While cancer survivorship care and research have been evolving in the western world over the past two decades,^11^ the concept of cancer survivorship remains new in China, and related research has been fragmented.

To fill this knowledge gap, this study provides an overview of research addressing various aspects of survivorship for childhood cancer survivors in China in the scientific literature published in English or Chinese language. We also discussed implications for childhood cancer care delivery and avenues for future research. The study was deemed exempt by the Ethics Committee of the Center for Health Management and Policy Research at Shandong University prior to commencing this study.

## 2 DOMAINS OF CANCER SURVIVORSHIP

To conceptualize cancer survivorship, we focused on key domains adapted from the Cancer Survivorship Care Quality Framework by Nekhlyudov et al.^12^

**FIGURE 1:**
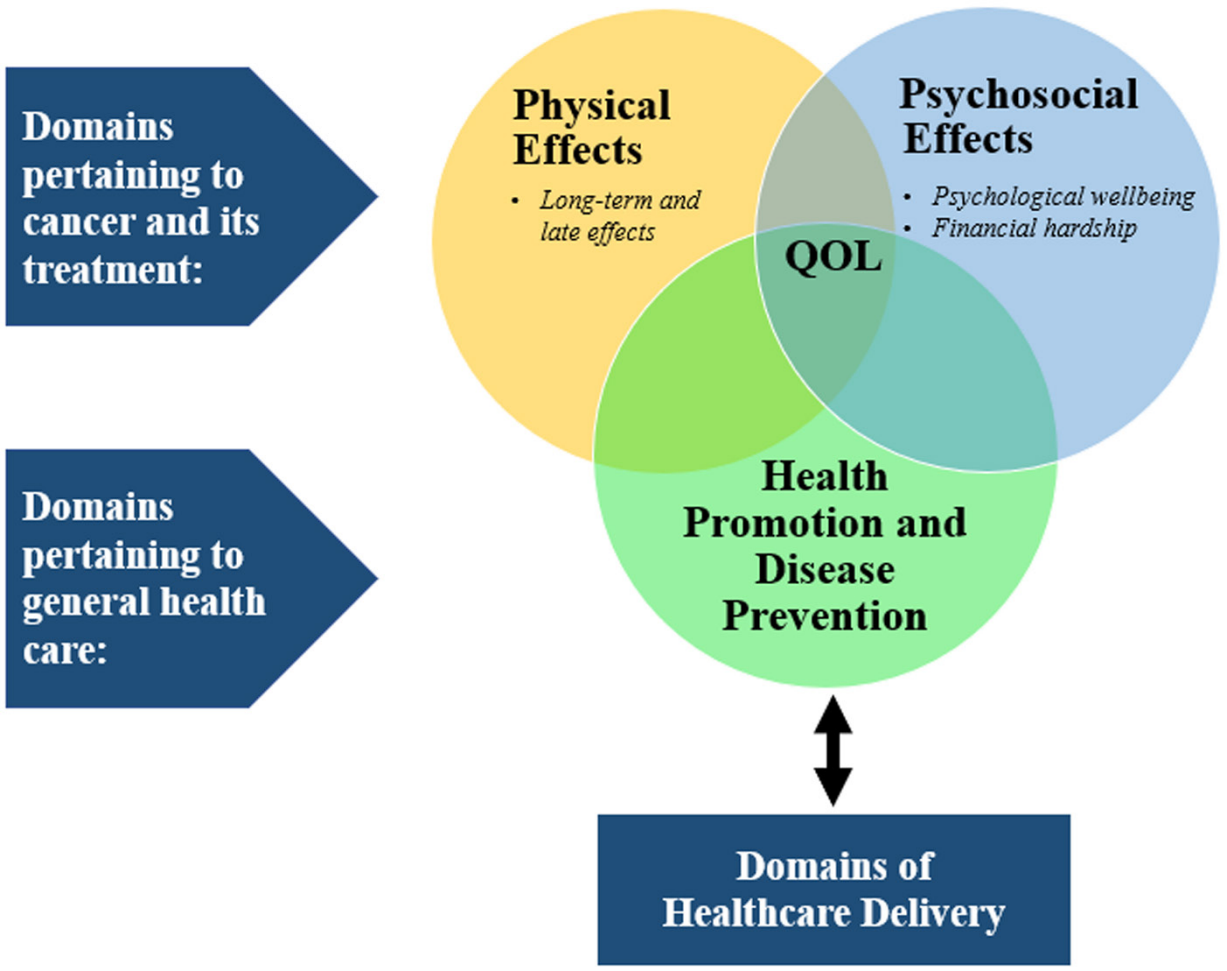
Domains of cancer survivorship. Notes. Adapted from the Cancer Survivorship Care Quality Framework by Nekhlyudov (2019). QOL, quality of life.

A major domain pertains to cancer and its treatment, including surveillance of physical sequelae—also called long‐term and late effects—and psychosocial effects of cancer diagnosis and treatment (Figure 1). Late effects (e.g., second cancers, cardiovascular diseases, and lung diseases) often last or occur months or years after cancer treatment is completed.^13^ Indicators of psychosocial effects commonly include psychologic well‐being (e.g., distress) and financial hardship.^12^, ^14^ Financial hardship is often measured in three subdomains: material conditions, psychological responses, and coping behaviors.^15^

Another domain of cancer survivorship pertains to general health care, including health promotion and disease prevention. Indicators in this domain often include lifestyle behaviors (e.g., alcohol consumption) and preventive services use (e.g., vaccination).^12^ Furthermore, cancer survivorship is also influenced by domains of healthcare delivery (e.g., survivorship care workforce, patient‐provider communication and decision‐making, and patients/caregivers experience).^12^

All the domains described above ultimately impact survivors' health outcomes including quality of life (QOL). QOL is an individuals' perception of their position in life in the context of their culture and value systems and in relation to their goals, expectations, standards, and concerns.^16^ QOL is multidimensional, and children's QOL is often measured in the physical, emotional, psychological, and social domains.

We used domains derived from the Cancer Survivorship Care Quality Framework^12^ as a guide to search and select literature (detailed in Appendix S1, S2) and synthesize findings from Chinese childhood cancer survivor populations. Below we presented highlights from studies published between January 1, 2000 and June 30, 2021 in English^17^, ^18^, ^19^, ^20^, ^21^, ^22^, ^23^, ^24^, ^25^, ^26^, ^27^, ^28^, ^29^, ^30^, ^31^, ^32^, ^33^, ^34^, ^35^, ^36^, ^37^, ^38^, ^39^, ^40^, ^41^, ^42^ or Chinese language^43^, ^44^, ^45^, ^46^, ^47^, ^48^, ^49^, ^50^, ^51^, ^52^, ^53^, ^54^, ^55^, ^56^, ^57^, ^58^, ^59^ (Table 1, Appendix S3) to shed light on the gaps in previous literature and opportunities for future research.

**TABLE 1:** Summary of articles on childhood cancer survivorship in China

Survivorship outcome	Citation [Reference #]	Place	Study design	Sample size	Study follow‐up period	Age range at study/interview	Cancer type
English literatures							
Long‐term and late effects of cancer treatment	Khalil 2019^17^	Shanghai	Hospital‐based retrospective cohort	86 survivors	Median 7 years since diagnosis	median follow‐up 84 months (range 24–120 months)	Medulloblastoma
	Cheung 2011^18^	Hong Kong	Hospital‐based case–control	36 survivors and 20 controls	1+ years off treatment	15.6 ± 5.5 yearsRange is not specified	Leukemia
	Yu 2013a^19^	Hong Kong	Hospital‐based Case–control	53 survivors and 38 controls	1+ years off treatment	18.6 ± 5.1 yearsRange is not specified	Acute lymphoblastic leukemia, acutemyeloid leukemia, osteosarcoma, Burkitt lymphoma, Hodgkin lymphoma, non‐Hodgkin lymphoma,synovial sarcoma, neuroblastoma and hepatoblastoma
	Yu 2013b^20^	Hong Kong	Hospital‐based Case–control	32 survivors and 28 controls	1+ years off treatment	19.3 ± 5.4 yearsRange is not specified	Not specified
	Li 2019a^21^	Hong Kong	Hospital‐based Case–control	83 survivors and 42 controls	5+ years off treatment	15+ years	Acute lymphoblastic leukemia, Non‐Hodgkin lymphoma, Acute myeloid leukemia, Osteosarcoma, Hodgkin lymphoma, Wilm's tumor,Peripheral primitive neuroectodermal tumor, Ewing Sarcoma, Hepatoblastoma
	Li 2019b^22^	Hong Kong	Hospital‐based Case–control	103 survivors and 61 controls	5+ years off treatment	25.6 ± 6.1 yearsRange is not specified	Acute lymphoblastic leukemia, Non‐Hodgkin's lymphoma, Acute myeloid leukemia, Wilms' tumor,Hodgkin's lymphoma, Osteosarcoma, Ewing sarcoma, Clear cell sarcoma of kidney, Ganglioneuroblastoma, Hepatoblastoma, Neuroblastoma, Peripheral primitive neuroectodermal tumor
	Xie 2018^23^	Guangzhou, Guangdong Province	Hospital‐based retrospective cohort	90 survivors	2.6–9.6 years after diagnosis; off treatment	Not specified	Nasopharyngeal carcinoma
	Lu 2019^24^	Guangzhou, Guangdong Province	Hospital‐based retrospective cohort	94 survivors	5–27 years (median 10 years) after completion of treatment	< 18 years	Nasopharyngeal carcinoma
	Chung 2014^25^	Hong Kong	Hospital‐based cross‐sectional	128 survivors	6+ months after completion of treatment	9–16 years	Leukemia, Lymphoma, Brain tumor, Osteosarcomas, Kidney tumor, Germ‐cell tumor
	Peng 2021^26^	Hong Kong	Hospital‐based cross‐sectional	152 survivors	2+ years off treatment	23.5 ± 7.2 years	Acute lymphoblastic leukemia
	Yang 2021a^27^	Hong Kong	Hospital‐based cross‐sectional	200 survivors	10+ years off treatment	Adult survivors: 26.9 ± 6.4 years;pediatric survivors: 11.1 ± 3.6 years	Hematological malignancy, Leukemia, Lymphomas, CNS tumor, Neuroblastoma Retinoblastoma, Renal tumor, Hepatic tumor, Bone tumor, Soft tissue sarcomas, Germ cell tumor, Others
Psychological wellbeing	Ng 2019^35^	Hong Kong	Hospital‐based cross‐sectional	200 Survivors	3+ years off treatment	25.4 ± 5.57 years	hematological cancer, acute lymphoid leukemia, acute myeloid leukemia, Hodgkin lymphoma, Wilm's tumor, osteosarcoma, neuroblastoma and others
	Yuen 2014^28^	Hong Kong	Non‐Government Organization‐based cross‐sectional	89 survivors	In remission	17.2–31.3 years	Not specified
	Li 2013^29^	Hong Kong	Hospital‐based cross‐sectional	137 survivors and 245 controls	6+ months after completion of treatment	9 to 16 years	Leukemia, Lymphoma, Brain tumor, Osteosarcoma,Kidney tumor, Germ cell tumor
Psychological wellbeing and quality of life	Cheung 2019a^31^	Hong Kong	Hospital‐based cross‐sectional	157 survivors	2+ months after completion of treatment	7 to 16 years	Brain cancer and other cancers
	Chan 2014^33^	Hong Kong	Hospital‐based cross‐sectional	614 survivors and 208 sibling controls	2+ y off treatment	16 to 39 years	Leukemia, Lymphoma, Bone and soft tissue cancers,Brain and CNS malignancies, and Others
	Cheung 2019b^32^	Hong Kong	Hospital‐based randomized trial	60 survivors	2+ months after completion of treatment	7–16 yMean = 12.53 (3.18) for experimental group;mean = 13.97 (3.26) for control group	Brain tumors
	Li 2018^36^	Hong Kong	Hospital‐based randomized trial	222 survivors	6+ after completion of treatment	9–16 years	Leukemia, Lymphoma, Brain tumor, Bone tumor, Neuroblastoma
Quality of life	Chung 2012^37^	Hong Kong	Hospital‐based cross‐sectional	153 survivors	6+ months after completion of treatment	9–16 years	Leukemia, Lymphoma, Brain tumor, Osteosarcoma,Kidney tumor, Germ cell tumor
	Zhang 2018^38^	Hangzhou, Zhejiang Province	Hospital‐based cross‐sectional	71 survivors and80 controls	1+ years after treatment	5 to 8 years	Etinoblastoma (RB)
	Ho 2019^39^	Hong Kong	Hospital‐based cross‐sectional	400 survivors	6+ after completion of treatment	7–18 years	Leukemia, Lymphoma, Brain tumor, Osteosarcoma,Kidney tumor, Germ cell tumor
	Yang 2021b^41^	Hong Kong	Hospital‐based cross‐sectional	80 survivors	off treatment and 5+ years after diagnosis	24.4 ± 6.5 years	Leukemia, Brain & CNS tumor, and other solid tumors (refers to germ cell tumor, osteosarcoma, soft tissue sarcoma, and lymphoma)
Health promotion, psychological wellbeing and quality of life	Chan 2020^34^	Hong Kong	Hospital‐based cross‐sectional	614 survivors and 208 sibling controls	2+ y off treatment	24.0 ± 5.1 years	Leukemia, Lymphoma, bone and soft tissue cancers,Brain and CNS malignancies, and Others
Health promotion and quality of life	Zheng 2021^42^	Guangzhou, Guangdong Province	Hospital‐based cross‐sectional	181 survivors	Off treatment and 2+ years after diagnosis	4–18 years	Leukemia, lymphoma, solid tumors
Quality of life and Caregiver wellbeing	Wang 2017^40^	Chengdu, Sichuan Province	Hospital‐based Case–control	217 survivors and95 controls	Currently not receiving treatment	0–2 years	Infantile hemangioma
Chinese literatures							
Long‐term and late effects of cancer treatment	Jiang 2000^43^	Shanghai	Hospital‐based cross‐sectional	31 survivors	3–14 years off treatment	10–20 years	Acute lymphoblastic leukemia
	Chen 2000^44^	Shanghai	Hospital‐based cross‐sectional	22 survivors	4–8.5 years (median 4 year 10 months) after remission	8–16 years	Acute leukemia
	Zhou 2006^47^	Suzhou, Jiangsu Province	Hospital‐based Case–control	30 survivors and 30 controls	3+ years after remission	6–34 years	Acute leukemia
	Zhao 2012^46^	Beijing	Hospital‐based Case–control	70 survivors and 36 controls	Median 64.3 months (15–131 months) since diagnosis	0.7–14.7 (median 4.5) years	Acute leukemia
	Fu 2017^45^	Shanghai	Hospital‐based Case–control	40 survivors and 40 controls	5+ years after remission	7–15 years	Acute lymphoblastic leukemia
	Zhang 2001^48^	Wenzhou, Zhejiang Province	Hospital‐based cross‐sectional	35 survivors	6–16 years after remission	8–28 years	Acute lymphoblastic leukemia
Psychological wellbeing	Wang 2009a^53^	Jinan, Shandong Province	Hospital‐based Case–control	19 survivors and 40 controls	5+ years after remission	7–16 years	Leukemia
	Wang 2010a^50^	Jinan, Shandong Province	Hospital‐based Case–control	20 survivors and 50 controls	5+ years after remission	7–16 years	Leukemia
	Wang 2010b^51^	Jinan, Shandong Province	Hospital‐based cross‐sectional	20 survivors and 30 controls	5+ years after remission	7–16 years	Leukemia
	Wang 2011^52^	Jinan, Shandong Province	Hospital‐based Case–control	20 survivors and 50 controls	5+ years after remission	7–16 years	Acute leukemia
	Sun 2006^54^	Suzhou, Jiangsu Province	Hospital‐based cross‐sectional	27 survivors	3+ years after remission	7–19 years	Acute leukemia
	Li 2021^55^	Beijing	Hospital‐based cross‐sectional	106 survivors	After remission	12–28 years	Leukemia
Quality of life	Fan 2007^56^	Zhanjiang, Guangdong Province	Hospital‐based Case–control	140 survivors and 80 controls	1+ years after remission	9–16 years	Lymphoma
	Liu 2018^57^	Chongqing	Hospital‐based Case–control	307 survivors	After remission	2–18 years	Leukemia
Caregiver wellbeing	Wang 2007^58^	Beijing	Hospital‐based Case–control	68 survivors and 122 controls	After remission	4–16 years	Leukemia
	Wang 2009b^49^	Jinan, Shandong Province	Hospital‐based Case–control	19 survivors and 40 controls	2–13 years (mean 5.26 years) off treatment	7–16 years	Acute leukemia
	Wang 2010a^50^	Jinan, Shandong Province	Hospital‐based Case–control	20 survivors and 50 controls	5+ years after remission	7–16 years	Leukemia
	Fu 2016^59^	Haikou, Hainan Province	Hospital‐based Case–control	106 survivors	Median 4+ years after remission	Not specified	Leukemia

## 3 RESEARCH ADDRESSING PHYSICAL LATE EFFECTS OF CANCER TREATMENT

Eleven studies published in English language and six studies published in Chinese examined the physical late effects of cancer treatment among childhood cancer survivors in China (Table 1).

We identified several studies conducted in Hong Kong, which reported cardiotoxic side effects of anthracycline chemotherapy among childhood cancer survivors who were off treatment for ≥1 year.^18^, ^19^, ^20^, ^21^, ^22^ Yu et al performed a Hong Kong‐based study of 53 adolescent and young adult survivors of childhood cancer, and demonstrated the impairment of global and regional myocardial deformation in three dimensions, reduced torsion, and systolic dyssynchrony after anthracycline therapy.^19^ In another Hong Kong‐based study focusing on 32 anthracycline‐treated survivors of childhood cancer, the same research team found reductions in left ventricular transmural circumferential strain and apical rotation gradients in survivors.^20^ In a similar study in Hong Kong, Cheung et al reported the impairment of left ventricular twisting and untwisting motion after anthracycline therapy in a cohort of 36 childhood survivors of acute lymphoblastic leukemia (ALL).^18^ In two recent Hong Kong‐based studies, Li et al investigated cohorts of 83 and 103 survivors of childhood cancer who had been off therapy for ≥5 years, and demonstrated low myocardial strain indices at imaging, abnormal left ventricular and right ventricular systolic functional reserve, and impairment of left ventricular diastolic functional reserve.^21^, ^22^ A more recent study in Hong Kong followed 152 young survivors of childhood ALL who were treated with chemotherapy and ≥2 years off treatment; this study found that, while the majority of the survivors had a normal cognitive and behavioral function, the impairments were higher than population norms. This study also found that chronic conditions developed after the cancer treatments were associated with multiple measures of behavior problems, such as executive dysfunction and attention problems.^26^ Although long‐term and late effects of cancer treatment are common among childhood cancer survivors, another study in Hong Kong found that, among 200 survivors at least 10 years post‐treatment, most were not able to accurately identify the late effects that they were at risk for,^27^ suggesting that improvements in health literacy of late effects are warranted among childhood cancer survivors.

We also identified several studies conducted in mainland China, which examined physical late effects among childhood cancer survivors. Several of these studies examined late effects associated with chemotherapy received by children diagnosed with acute leukemia (Table 1).^43^, ^44^, ^45^, ^46^, ^47^, ^48^ A range of 22–70 children from Suzhou, Wenzhou, Shanghai, or Beijing who were several years off treatment or after remission were included in the studies. These studies generally did not find evidence of late effects on growth, development, or endocrine function; however, a reduction in intelligence quotient was reported in two of the studies in Shanghai.^44^, ^45^ In addition, two studies in mainland China examined radiotherapy‐induced toxicity and late sequelae of complications. In a study examining 90 children diagnosed with nasopharyngeal carcinoma and treated at a cancer center in Guangzhou, Xie et al followed survivors for 2.6–9.6 years after their receipt of radiotherapy; they found that survivors had decreased pituitary heights and stunted linear growth.^23^ In another Guangzhou‐based study, Lu et al identified 94 survivors of childhood and adolescent nasopharyngeal carcinoma treated with radiotherapy and followed survivors for a median of 10 years.^24^ This study showed the presence of grade 1 and 2 complications (such as xerostomia, hearing loss, and neck fibrosis; graded according to the National Cancer Institute Common Terminology Criteria for Adverse Events version 3.0) in most survivors who received radiotherapy.^24^ This study also reported that the top 10 common long‐term sequela of radiotherapy included xerostomia, hearing loss, neck fibrosis, trismus, caries, dysphagia, impaired memory, tinnitus, lalopathy, and chronic otitis media, with a higher radiation dose associated with higher incidence of severe late sequelae.^24^ Lastly, in a Shanghai‐based study following 121 childhood medulloblastoma survivors treated with surgery, radiotherapy, and/or chemotherapy, Khalil et al reported growth suppression in 41.3% survivors, hearing loss in 24% survivors, visual disturbance in 15.7% survivors, neurologic toxicity (including poor concentration, poor memory, and learning difficulties) in 25% survivors, and secondary malignancy in 3.5% survivors.^17^

## 4 RESEARCH ADDRESSING PSYCHOLOGICAL WELLBEING

Eight studies published in English language and six studies in Chinese investigated psychological well‐being of childhood cancer survivors in China (Table 1).

The English articles identified in this domain were all based in Hong Kong.^28^, ^29^, ^30^, ^31^, ^32^, ^33^, ^36^ Using a convenient sample in an oncology clinic in Hongkong, Li et al interviewed 137 childhood cancer survivors ≥6 months after treatment completion (73% within 2 years post‐treatment).^29^ The study showed that childhood cancer survivors had higher risk for depressive symptoms and lower levels of self‐esteem, compared with peers without a cancer history.^29^ In a recent study of 77 childhood brain tumor survivors and 80 survivors of other childhood cancers, Li et al reported that brain tumor survivors had the poorest psychological outcomes, assessed by the number of depressive symptoms and the level of self‐esteem.^31^ In contrast, using convenient samples of 614 childhood cancer survivors who were off treatment for ≥2 years and 208 sibling controls, Chan et al found no difference in mental, social, or psychological wellbeing between survivors and their siblings.^33^ Using the same study subjects, Chan et al also found that unhealthy behaviors such as alcohol drinking and lacking cancer screenings were associated with higher psychological distress among survivors.^34^ In a study examining adult survivors of childhood cancers with original tumors not hormone‐dependent, Ng et al examined sexual function, an integral part of both physical and psychosocial wellbeing, and found that 24% survivors reported sexual functioning problems.^35^ They also found that survivors with non‐hematological cancers, those treated with surgery, and those with lower health‐related quality of life, lower self‐esteem, and higher levels of body image distress and depression symptoms were more likely to report sexual functioning problems.^35^

Psychological wellbeing of childhood cancer survivors has also been studied in mainland China. In a line of research conducted in Jinan, Shandong Province, Wang et al interviewed 20 child survivors of leukemia who were ≥5 years after remission and compared their psychological constructs with healthy children (Table 1).^50^, ^51^, ^52^, ^53^ They found that survivors had higher scores on somatization/panic, generalized anxiety, and social phobia and lower scores on self‐concept and happiness, compared with healthy controls, and survivors' psychological scores were correlated with parents' anxiety scores.^50^, ^51^, ^52^, ^53^ In another study of 27 child survivors of acute leukemia who were ≥3 years after remission in Suzhou, Jiangsu Province, Sun et al reported a high prevalence of learning anxiety (85.7%), a tendency toward being oversensitive and self‐blaming (21.4%), and a tendency toward isolation (7.1%) (Table 1).^54^ A recent study of 106 adolescent leukemia survivors in Beijing showed that disease risk, duration of drug withdrawal, and general self‐efficacy were the contributing factors of psychological resilience.^55^ Notably, we only identified one study investigating effective intervention strategies toward improving psychosocial outcomes of childhood cancer survivors.^32^

## 5 RESEARCH ADDRESSING QUALITY OF LIFE

Twelve studies published in English language and two studies published in Chinese examined QOL of childhood cancer survivors in China (Table 1).

Studies focusing on QOL of childhood cancer survivors were largely conducted in Hong Kong. In a telephone survey of historical patients treated in three Hong Kong hospitals who were ≥2 years off treatment, young adult survivors (aged 16–39 years) of childhood cancer had inferior health‐related quality of life (HRQOL) compared to their siblings in the physical health components.^33^ Among the survivors, older age, female gender, receipt of multiple treatments, and cancers of bone, soft tissue, and CNS cancer were associated with poorer HRQOL.^33^ In another series of studies, childhood cancer survivors aged 7–18 years who had completed cancer treatment for at least several months were interviewed in an outpatient clinic setting in Hong Kong.^30^, ^31^, ^37^, ^39^ These studies showed that greater symptoms of depression, greater occurrence and severity of fatigue, and a diagnosis of brain tumor were associated with poorer QOL. Another study in Hong Kong showed that alcohol drinking was associated with poorer QOL among young adult survivors of childhood cancers.^34^ In two recent randomized controlled trials conducted among Hong Kong children who had completed cancer treatment for at least several months, a one‐year music training program^32^ and a 4‐day per week adventure program^36^ effectively enhanced QOL for brain tumor survivors and survivors with fatigue symptoms, respectively. One recent study of young adult survivors of childhood cancers in Hong Kong examined life functioning, which is often considered a dimension of QOL,^60^ and found that brain tumor survivors and those treated with cranial radiation performed worse in social functioning and worker life functioning.^41^

In mainland China, studies on QOL of childhood cancer survivors have been sparse until recently. A Hangzhou‐based study followed childhood retinoblastoma survivors aged 5–8 years seen in an eye center, and showed that survivors' QOL were significantly lower than those of healthy children controls, especially in the dimensions of social and school functioning.^38^ The study further showed that bilateral eye disease, earlier age at diagnosis, and low degree of satisfaction with the artificial eyes were associated with worse QOL among survivors.^38^ A study conducted at the department of pediatric surgery in a hospital in Chengdu found that children with infantile hemangioma (<2 years off‐treatment) had comparable QOL with healthy controls in all functioning dimensions, except for physical symptoms.^40^ Among survivors, hemangioma size, tumor location, children's age, and parents' education level influenced QOL.^40^ A more recent study in Guangzhou found that physical and psychosocial domains of QOL in childhood cancer survivors were lower than the norm score assessed in healthy children.^42^ Additionally, pick‐eating was associated with lower QOL, and moderate‐to‐vigorous physical activity was associated with higher QOL among childhood cancer survivors.^42^

Only two eligible studies on QOL of childhood cancer survivors off treatment were identified in the literature published in Chinese language, where QOL of 140 child survivors of lymphoma and 80 healthy controls in Zhanjiang and Shenzhen were included.^56^ One study showed that compared with healthy controls, survivors 1‐year post‐remission had worse scores in the domains of role function, social function, economic status, and general health; additionally, survivors 5‐year post‐remission still had worse scores in social function and general health. Another study from Chongqing explored the effect of continuous nursing based on short distance communication mode on the QOL of 307 children with leukemia, and this mode effectively reduced the incidence of infection and improved the quality of life in these survivors.^57^

## 6 RESEARCH ADDRESSING HEALTH PROMOTION

We identified two studies in English that examined health promotion among childhood cancer survivors in China (Table 1).

Using survey among 614 survivors and 208 sibling controls in Hong Kong, Chan et al found that survivors were less likely to drink alcohol and to have pap smear tests or breast examinations compared to their siblings.^34^ Another study led by Zheng was conducted in Guangzhou, one of the major cities insouthernh mainland China, and found that few Chinese cancer survivors engaged in unhealthy dietary behaviors, such as frequent soft drinks and fast food consumptions; however, many of them were picky eaters and did not meet milk intake and physical activity recommendations.^42^ Both studies found a link between unhealthy behaviors and worse QOL.^34^, ^42^

## 7 RESEARCH ADDRESSING CAREGIVER WELLBEING

We identified one study in English and four studies in Chinese that examined caregivers' well‐being post cancer treatment in China; all these studies were conducted in mainland China (Table 1).

In a study conducted in Chengdu, Sichuan Province, QOL of parents of 237 children with infantile hemangioma was correlated with mother's education level and patients' QOL.^40^ In another study comparing families of 68 children in remission after treatment of leukemia in 4 tertiary hospitals in Beijing with 122 healthy children, Wang et al found that families with a survivor had higher family cohesion and adaptability but less balanced family type.^58^ Additionally, in a study including 202 parents of 106 leukemia children in Haikou, Hainan Province, intensive health education reduced the degree of anxiety among parents in the observation group, compared with those receiving routine health education in the control group.^59^ Lastly, in two studies in Jinan, Shandong Province, parents of 19 and 20 children with leukemia who were ≥5 years after remission reported higher depression and anxiety scores, compared with parents of 40 and 50 healthy children, respectively.^49^, ^50^

## 8 RESEARCH ADDRESSING FINANCIAL HARDSHIP

Despite a thorough search of the literature, we did not find any study conducted in China focusing on the financial hardship of childhood cancer survivors and families in the post‐treatment period. Notably, there were five studies published in Chinese language that estimated care costs associated with cancer treatment among patients actively receiving cancer therapies.^61^, ^62^, ^63^, ^64^, ^65^ As costly cancer therapy is a strong driver of financial hardship during survivorship, we summarized the cost estimation in these studies in Appendix S4.

## 9 DISCUSSION

In this overview of research addressing childhood cancer survivorship in China, we found that previous literature primarily focused on physical sequelae of cancer therapy. Common physical late effects identified among childhood cancer survivors in China included cardiovascular diseases, second cancers, neurological and cognitive problems, and growth and hormone problems, with wide variation by treatment modality and cancer type.^17^, ^18^, ^19^, ^20^, ^21^, ^22^, ^23^, ^24^, ^25^, ^26^, ^44^, ^45^ A handful of studies explored the psychosocial effects of cancer therapy among childhood cancer survivors in China, where depression, anxiety, psychological distress, low self‐esteem, and behavioral problems were found to be common psychological problems.^28^, ^29^, ^30^, ^33^, ^50^, ^51^, ^52^, ^53^, ^54^ Childhood cancer survivors often experienced elevated rates of psychological problems and poorer QOL compared with healthy counterparts, with the magnitude of the difference varying by cancer type, age at diagnosis, and dimensions of outcome measures. Among survivors, adverse psychosocial outcomes were correlated with survivors' demographic characteristics (e.g., age, gender), clinical factors (e.g., cancer type, survival time, treatment modalities), lifestyle factors (e.g., physical activity), and parental factors.^29^, ^33^, ^37^

These findings highlight several gaps in the existing literature and opportunities to further study childhood cancer survivorship in the Chinese population. According to the Cancer Survivorship Care Quality Framework,^12^ prior studies have exclusively focused on domains pertaining to cancer and its treatment. Notably, these studies were largely based on a single institution, cross‐sectional design, convenient samplings with relatively small sample sizes, and restricted geographic area (i.e., Hong Kong or economically developed metropolitan cities of mainland China), potentially limiting the generalizability of findings. Further, very few studies had long‐term follow‐up after survivors completed cancer therapy; the median follow‐up time was less than a decade. Consequently, most studies focused on young survivors in their childhood or adolescence. Additionally, evidence on intervention strategies that improve long‐term physical and psychosocial sequelae of cancer and its treatment is sparse. These are also common limitations in the existing studies focusing on childhood cancer survivorship in other resource‐limited countries.^66^, ^67^ Together, future population‐based studies that allow longitudinal follow‐up of long‐term survivors of childhood cancer and new initiatives that enhance data infrastructure are needed in China to advance understanding of long‐term consequences of childhood cancer and its treatment, and to inform interventions toward prevention and early detection of late effects.

The well‐being and health of caregivers has been an active area of research in western countries;^68^ however, relevant data are lacking in China. Only four studies based in China examined caregivers of childhood cancer survivors, and generally found deteriorating mental health or family function problems of parents taking care of a child survivor;^40^, ^49^, ^50^, ^58^ yet, few examined caregivers' QOL nor the risk factors of their health and well‐being. Thus, more analyses are needed to assess various outcome domains, particularly QOL, among survivors' caregivers in China, and to identify effective interventions toward improving caregivers' health and well‐being. Several China‐based studies have explored effective strategies while children were under treatment, including tailored nursing models, mutual help groups for parents, or mHealth supportive care intervention,^69^, ^70^, ^71^ to buffer the effect of cancer diagnosis and treatment on their caregivers. These strategies could potentially be applied to caregivers of survivors off treatment.

With rising costs of cancer care, medical financial hardship and the consequent non‐medical financial sacrifices have become a major concern for survivors and families.^15^, ^72^, ^73^ To date, however, no studies have examined the financial hardship of long‐term survivors of childhood cancers and their families in China. In the existing studies focusing on costs of cancer treatments the and financial burden on families with a child actively receiving therapies, all used relatively small samples of leukemia children from a single hospital, with wide variation and limited generalizability (Appendix S4). In the U.S. and European countries, a series of studies—based on longitudinal follow‐up of 5‐year survivors or national survey databases—have shown an elevated risk for facing high out‐of‐pocket medical expenses, having difficulties with obtaining health insurance and paying medical bills, considering filing for bankruptcy, and lacking the ability to work among adult survivors of childhood cancer as compared to siblings or healthy controls.^74^, ^75^ It is critical to extend this research to China. Notably, when studying financial hardship for the growing population of childhood cancer survivors and their families in China, unique challenges stemming from the healthcare system in the country should be considered. Particularly, despite the recently achieved “universal health coverage” in China, out‐of‐pocket expenses still account for nearly 40% of total medical expenses.^76^ In addition, pediatric oncology care resources are concentrated only in a few metropolitan cities, leading to considerable costs associated with transportation and lodging for survivors residing in other areas.

There are only two recent studies in China on health promotion or disease prevention among survivors off childhood cancer treatment, an important area that merits future research. Relevant research topics would include assessment of lifestyle behaviors (e.g., smoking, alcohol use, illicit drug use, and physical activity), vaccination, and adherence to surveillance for late effects, which are vital indicators for cancer survivorship quality.^12^ Data from western countries have demonstrated that young cancer survivors are often inactive,^77^ continue to smoke,^78^ and have higher rates of alcohol use^79^ and less HPV vaccine initiation than those without cancer.^80^ Such vulnerabilities may be exacerbated among survivors in China, where smoking and harmful drinking behaviors are highly prevalent,^81^ and the fee‐for‐service delivery system often incentivizes treatments over preventive care;^82^ but this question has yet to be explored in the country.

Importantly, we are not aware of any study based in China that examine access to and delivery of survivorship care. This finding may be explained, at least partially, by the fact that in China, survivorship care has not been fully accepted as standard care for cancer survivors.^83^ As recognized in an expert survey of a panel of Chinese Children Cancer Group, there are major barriers to implementing survivorship programs, including unawareness of the health issues related to cancer therapy and concerns about privacy issues in survivors and families, as well as a lack of support and resources to provide follow‐up care for clinicians.^84^ Furthermore, in North American countries, risk‐based survivorship care is recommended for all childhood cancer survivors,^85^ and evidence‐based guidelines have been developed for the surveillance of late and long‐term effects.^86^, ^87^ However, in China, a lack of standardized guidance for monitoring late effects in survivors is another barrier to implementing cancer survivorship programs.^84^ To inform survivorship care practice in China, more China‐based studies are urgently needed to understand childhood cancer survivors' needs—including their awareness of potential long‐term health issues and risks—and Chinese pediatric oncologists' perceptions about the barriers and facilitators to implementing survivorship care. This line of future research will be a critical step toward developing survivorship care models that are practical and acceptable in China's unique historical and cultural context.

This is the very first, comprehensive overview of childhood cancer survivorship in the Chinese population on the basis of the scientific literature in English or Chinese languages. Our findings highlight the urgent need for research to address the gaps in knowledge about childhood cancer survivorship in China, and to develop evidence‐based consensus and guidelines for survivorship care practice and delivery, in order to meet the complex physical and psychosocial needs of the large and growing childhood cancer survivor population in the country.

## AUTHOR CONTRIBUTIONS

Xu Ji, Xiaojie Sun, and Xuesong Han: Conceptualization, visualization, methodology, writing—original draft, writing—review, and editing. Jun Sun, Xinyu Liu, Ziling Mao, Wenjing Zhang, and Jinhe Zhang: Methodology, literature review and organization, writing—review, and editing.

## CONFLICT OF INTEREST

The authors have no conflicts of interest to disclose.

## Supporting information


Appendix

## Data Availability

Data sharing is not applicable to this article as no data were created or analyzed in this study.
