# Supplementary material for: Childhood cancer survivorship in China: An overview of the past two decades
Source: Cancer Med. 2022 May 22;11(23):4588–601. doi: 10.1002/cam4.4831 (PMC9741979; doi:10.1002/cam4.4831)
Supplement: Supplementary file 1 — Appendix [file CAM4-11-4588-s001.docx]

**Appendix S1**. Search Strategy and Selection Criteria

We focused on studies published from January 1, 2000 to June 30, 2021. For the literature in English language, we used the following search strategy for full text search in PubMed/MEDLINE, CINAHL, Scopus, and Embase: ("China" OR "Chinese") AND ("childhood" OR "children" OR "pediatric" OR "pediatrics" OR "adolescent" OR "adolescence" OR "teenager" OR "teenage") AND ("cancer" OR "malignancy" OR "leukemia" OR "neoplasms" OR "tumor" OR "oncology" OR "therioma" OR "adenoma" OR "lymphoma" OR "melanoma" OR "multiple myeloma" OR "mesothelioma" OR "kaposi sarcoma") AND ("survivor" OR "survivors" OR "survivorship" OR "insurance" OR "access to care" OR "care communication" OR "financial" OR "employment" OR "function" OR "infertility" OR "pain" OR "comorbidity" OR "comorbid" OR "complication" OR "dysfunction" OR "quality of life" OR "distress" OR "social" OR "caregivers"), and identified 1,428 articles.

Further screening by carefully reviewing the titles and abstract resulted in 60 articles among Chinese childhood cancer patients, survivors, families, and caregivers that were deemed relevant with original research data and were selected for full text review. References cited by these articles were also reviewed, and two additional articles were identified. From the 62 articles for full text review, we excluded 10 qualitative studies and 26 studies focusing on childhood cancer patients under active treatment, resulting in a total of 26 articles in English included in this review (also see Table 1).

The same criteria were used to identity the literature in Chinese language from the CNKI (<https://www.cnki.net/>) and Wanfang (<http://www.wanfangdata.com/>) databases. The CNKI and Wanfang databases cover almost all peer-reviewed journal articles, Master’s theses, and doctoral dissertations published in Chinese and across all research topics. After selection with similar screening procedures, 17 articles published in Chinese peer-reviewed journals were included in our review (Table 1).

**Appendix S2**. Study selection flowchart

**
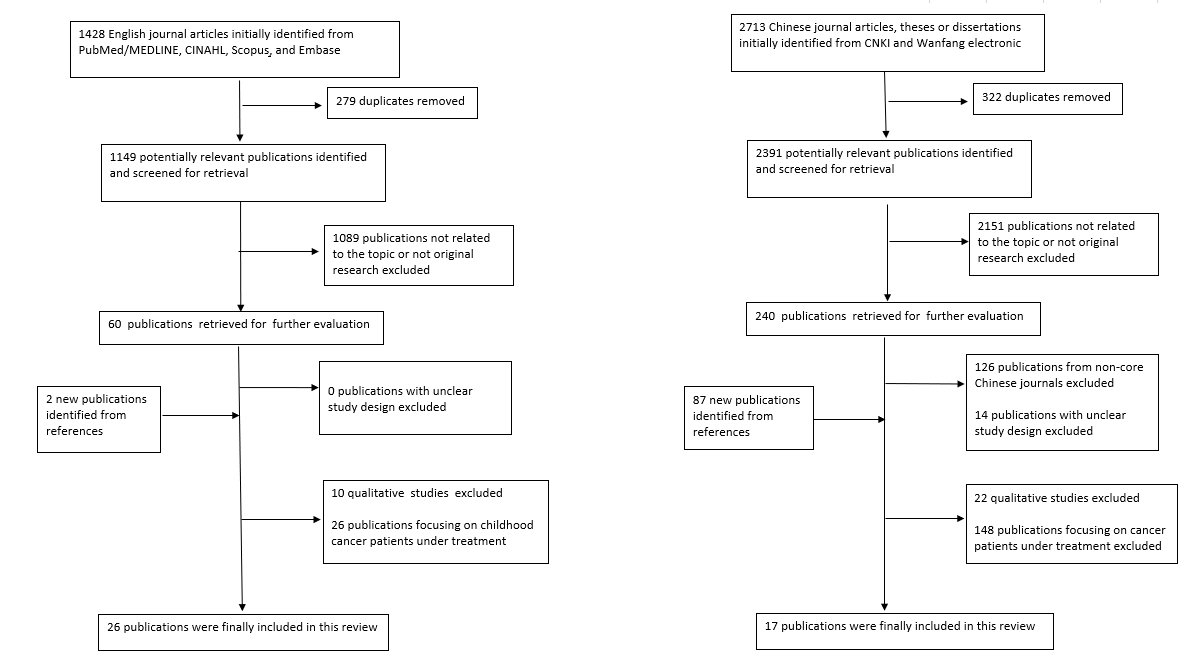
**

**Appendix S3**. Chinese references and citations translated into English

| **作者** | **发表年份** | **中文参考文献** | **Translation for Citation** |
| --- | --- | --- | --- |
| 常锐霞 | 2015 | 常锐霞,陈志鹏,席亚明, 等. 2003-2012年甘肃省白血病患儿住院治疗直接经济负担调查[J].中国全科医学,2015,18(5):569-572. DOI:10.3969/j.issn.1007-9572.2015.05.019. | Chang RX, Chen ZP,Xi YM, et al. Analysis of Direct Economic Burden in Hospitalized Leukemia Children from 2003 to 2012, Gansu Province.  *Chinese General Practice.* 2015;18(5):569-572. DOI:10.3969/j.issn.1007-9572.2015.05.019. |
| 陈静 | 2000 | 陈静,顾龙君,姚慧玉.22例小儿急性白血病长期无病生存质量的评估[J].中华儿科杂志,2000,38(2):111-112. DOI:10.3760/j.issn:0578-1310.2000.02.016. | Chen J, Gu LJ, Yao HY. Evaluation of long-term disease-free quality of life in 22 children with acute leukemia. *CHINESE JOURNAL OF PEDIATRICS*. 2000;38(2):111-112. DOI:10.3760/j.issn:0578-1310.2000.02.016. |
| 樊志明 | 2007 | 樊志明,郭伟雄.恶性淋巴瘤长期生存患儿生存质量调查[J].华南国防医学杂志,2007,21(4):50-51. DOI:10.3969/j.issn.1009-2595.2007.04.019. | Fan ZM, Guo WX. Investigation into Quality of Life in Long-term Survival Children with Malignant Lymphomas. *MILITARY MEDICAL JOURNAL OF SOUTH CHINA.* 2007;21(4):50-51. DOI:10.3969/j.issn.1009-2595.2007.04.019. |
| 符浩 | 2016 | 符浩,李春燕,周丽,等. 集中型健康教育模式对白血病患儿家属知信行及心理状况的影响. 中国小儿血液与肿瘤杂志, 2016,21(4):198-210. DOI: 10.3969/j.issn.1673-5323.2016.04.006 | Fu H, Li CY, Zhou L, et al. Effects of intensive health education KAP and psychological status of leukemia patients' families. *J China Pediatr Blood Cancer*. 2016,21(4):198-210. DOI: 10.3969/j.issn.1673-5323.2016.04.006 |
| 傅晓燕 | 2017 | 傅晓燕,谢晓恬,赵焱.长期无病生存急性淋巴细胞白血病儿童神经认知功能及影响因素的研究[J].中国当代儿科杂志,2017,19(8):899-903. DOI:10.7499/j.issn.1008-8830.2017.08.010. | Fu XY, Xie XT, Zhao Y. Neurocognitive function of children with acute lymphoblastic leukemia and long-term disease-free survival and related influencing factors. *Chinese Journal of Contemporary Pediatrics*. 2017;19(8):899-903. DOI:10.7499/j.issn.1008-8830.2017.08.010. |
| 黄雪丽 | 2019 | 黄雪丽,张慧敏,杨苗苗, 等.急性白血病患儿家庭经济负担和客观社会支持调查研究[J].卫生经济研究,2019,36(7):38-40. | Huang XL, Zhang HM, Yang MM, et al. Investigation on Family Economic Burden and Objective Social Support of Children with Acute Leukemia.  *Health Economics Research*. 2019;36(7):38-40. |
| 蒋慧 | 2000 | 蒋慧,陆正华,景虹.31例急性白血病停药后生存质量的调查[J].中华儿科杂志,2000,16(12):772. DOI:10.3760/j.issn:0578-1310.2000.12.014. | Jiang H, Lu ZH, Jing H. Quality of life in 31 patients with acute leukemia after drug withdrawal. *CHINESE JOURNAL OF PEDIATRICS.* 2000;16(12):772. DOI:10.3760/j.issn:0578-1310.2000.12.014. |
| 姜璎硕 | 2018 | 姜璎硕,甄慧宇,张明慧, 等. 基于IKAP模式的个体化护理对白血病患儿家属焦虑抑郁状况的影响[J].国际精神病学杂志,2018,45(4):765-768. | Jiang YS, Zhen HY, Zhang MHJ, et al. Influence of Individualized Nursing Based on IKAP Model on Anxiety and Depression Status of Family Members of Children with Leukemia. *Journal of International Psychiatry.* 2018;45(4):765-768. |
| 李慧君 | 2021 | 李慧君,王旭梅,吴心怡,等. 青少年白血病幸存者心理韧性现状及影响因素分析[J]. 中华现代护理杂志,2021,27(25):3432-3437. DOI:10.3760/cma.j.cn115682-20201221-06807 | Li HY, Wang XM, Wu XY, et al. Current status and influencing factors of psychological resilience in adolesent leukemia survivors. *Chinese Journal of Morden Nursing*. 2021, 27(25):3432-3437. DOI：10.3760/cma.j.cn115682-20201221-06807 |
| 刘洋 | 2018 | 刘洋,马琳玉,莫霖,等. 基于信息化的短距离沟通方式在白血病患儿延续护理中的应用 [J]解放军护理杂志, 2018,35(13):49-53. DOI: 10.3969/j.issn.1008-9993.2018.13.012 | Liu Y, Ma LY, Mo L, et al. Application of short distance communication mode on continuous nursing for children with leukemia. *Nursing Journal of Chinese People's Liberation Army*. 2018,35(13):49-53. DOI: 10.3969/j.issn.1008-9993.2018.13.012 |
| 任益炯 | 2015 | 任益炯,季庆英,张佳妮, 等. 儿童急性白血病患者家庭负担调查[J].解放军医院管理杂志,2015,(1):19-21. | Ren YJ, Ji QY, Zhang JN, et al. The Disease -Related Burden of Families with Children with Acute Lymphoblastic Leukemia. *Hospital Administration Journal of Chinese People’s Liberation Army.* 2015;(1):19-21. |
| 孙伊娜 | 2006 | 孙伊娜,柴忆欢,何海龙, 等. 儿童急性白血病长期持续化疗后智力与心理行为的调查分析[J].中国小儿血液与肿瘤杂志,2006,11(6):297-300. DOI:10.3969/j.issn.1673-5323.2006.06.003. | Sun YN, Chai YH, He HL, et al. Investigation and analysis of the intelligence and mentality in children with acute leukemia after long-term chemotherapy. *JOURNAL OF CHINA PEDIATRIC BLOOD AND CANCER.* 2006;11(6):297-300. DOI:10.3969/j.issn.1673-5323.2006.06.003. |
| 王红美 | 2009 | 王红美,何守森,陈力军, 等.长期无病生存白血病儿童情绪、自我意识及个性特征的对照研究[J].中华行为医学与脑科学杂志,2009,18(5):412-414. DOI:10.3760/cma.j.issn.1674-6554.2009.05.011. | Wang HM, He SS, Chen LJ, et al. A controlled study on the psychological characteristics of long-term survivors of childhood leukemia. *CHINESE JOURNAL OF BEHAVIORAL MEDICINE AND BRAIN SCIENCE.* 2009;18(5):412-414. DOI:10.3760/cma.j.issn.1674-6554.2009.05.011. |
| 王红美 | 2009 | 王红美,陈力军,陈伟, 等.白血病患儿情绪自我意识及其父母情绪特点的对照研究[J].中国实用儿科杂志,2009,24(10):791-794. | Wang HM, Chen LJ, Chen W, et al. A controlled study on the psychological characteristics of children with leukemia and their parents. *CHINESE JOURNAL OF PRACTICAL PEDIATRICS*. 2009;24(10):791-794. |
| 王红美 | 2010 | 王红美,陈力军,高飞, 等.白血病患儿及其父母心理特点调查分析[J].中国小儿血液与肿瘤杂志,2010,15(4):152-156,166. DOI:10.3969/j.issn.1673-5323.2010.04.004. | Wang HM, Chen LJ, Gao F, et al. Study on the psychological characteristics of children with leukemia and their parents. *JOURNAL OF CHINA PEDIATRIC BLOOD AND CANCER.* 2010;15(4):152-156,166. DOI:10.3969/j.issn.1673-5323.2010.04.004. |
| 王红美 | 2010 | 王红美,任万华,张玉梅, 等. 初诊及长期无病生存白血病儿童情绪、自我意识及个性特征的调查分析[J].临床儿科杂志,2010,28(5):433-437. DOI:10.3969/j.issn.1000-3606.2010.05.008. | Wang HM, Ren WH, Zhang YM, et al. A controlled study on the psychological characteristics of long-term survivors of childhood leukemia and newly diagnosed leukemia patients. *JOURNAL OF CLINICAL PEDIATRICS.* 2010;28(5):433-437. DOI:10.3969/j.issn.1000-3606.2010.05.008. |
| 王红美 | 2011 | 王红美,张玉梅,陈伟, 等. 父母的焦虑、抑郁对白血病患儿情绪及自我意识的影响[J].肿瘤研究与临床,2011,23(7):483-486. DOI:10.3760/cma.j.issn.1006-9801.2011.07.015. | Wang HM, Zhang YM, Chen W, et al. Effects of parental anxiety and depression on the feelings and self-concept of children with leukemia. *CANCER RESEARCH AND CLINIC.* 2011;23(7):483-486. DOI:10.3760/cma.j.issn.1006-9801.2011.07.015. |
| 王俊 | 2019 | 王俊,张福来.医院感染对住院费用的影响研究——以儿童急性淋巴细胞白血病为例[J].卫生经济研究,2019,36(1):26-28. | Wang J, Zhang FL. Study on the Effect of Nosocomial Infection on Hospitalization Expenses——Taking Children with Acute Lymphoblastic Leukemia as An Example. *Health Economics Research*. 2019;36(1):26-28. |
| 王蕾 | 2007 | 王蕾,洪黛玲.缓解期白血病患儿家庭亲密度和适应性的调查研究[J].护理学杂志,2007,22(23):25-26. DOI:10.3969/j.issn.1001-4152.2007.23.012. | Wang L, Hong DL. Investigation on Family Cohesion and Adaptability of Leukemia Children During Remission Period. *JOURNAL OF NURSING SCIENCE.* 2007;22(23):25-26. DOI:10.3969/j.issn.1001-4152.2007.23.012. |
| 王小苗 | 2018 | 王小苗,范菱.以家庭为中心的护理在白血病患儿中的应用及对家属希望水平、患儿心理韧性和生活质量的影响[J].检验医学与临床,2018,15(24):3752-3755. DOI:10.3969/j.issn.1672-9455.2018.24.031. | Wang XM, Fan L. Application of family centered nursing in children with leukemia and its influence on hope level of family members, psychological resilience and quality of life of children. *Laboratory Medicine and Clinic.* 2018;15(24):3752-3755. DOI:10.3969/j.issn.1672-9455.2018.24.031. |
| 吴丽芬 | 2014 | 吴丽芬,刘恋,何娇, 等. 建立白血病患儿家长支持系统的效果评价[J].护理研究,2014,28(8):964-965. DOI:10.3969/j.issn.1009-6493.2014.08.029. | Wu LF, Liu L, He J, et al. Effect evaluation on establishment of support system of parents of children with leukemia. *Chinese Nursing Research*. 2014;28(8):964-965. DOI:10.3969/j.issn.1009-6493.2014.08.029. |
| 章佳珠 | 2001 | 章佳珠,阮积晨,李原, 等. 儿童急性淋巴细胞性白血病长期生存生活质量的调查[J].温州医学院学报,2001,31(4):232-233. DOI:10.3969/j.issn.1000-2138.2001.04.012. | Zhang JZ, Ruan JC, Li Y, et al. A survey of the long-term survival quality for children with acute lymphocytic leukemia. *JOURNAL OF WENZHOU MEDICAL COLLEGE.* 2001;31(4):232-233. DOI:10.3969/j.issn.1000-2138.2001.04.012. |
| 赵国强 | 2020 | 赵国强.白血病患儿疾病经济负担及慈善医疗救助补偿效果研究—以 Z 项目为例[D].山东:山东大学,2020. | Zhao GQ. Study on the economic burden of leukemia children and the compensation effect of charity medical assistance -- a case study of Z project [M], Shandong University; 2020. |
| 赵卫红 | 2012 | 赵卫红,华瑛,卢新天, 等. 急性淋巴细胞白血病患儿长期疗效分析[J].实用儿科临床杂志,2012,(3):192-193,202. DOI:10.3969/j.issn.1003-515X.2012.03.015. | Zhao WH, Hua Y, Lu XT, et al. Analysis of Follow - Up of Therapeutic Effectiveness in Children with Acute Lymphoblastic Leukemia. *Journal of Applied Clinical Pediatrics*. 2012;(3):192-193,202. DOI:10.3969/j.issn.1003-515X.2012.03.015. |
| 周家兴 | 2006 | 周家兴,柴忆欢.30例小儿急性白血病长期无病生存的生长发育及内分泌状态分析[J].中国小儿血液与肿瘤杂志,2006,11(5):248-251. DOI:10.3969/j.issn.1673-5323.2006.05.003. | Zhou JX, Chai YH. Growth, Analysis of growth and development and endocrinosity in 30 acute leukemic children of long-term disease-free survival.  *JOURNAL OF CHINA PEDIATRIC BLOOD AND CANCER*. 2006;11(5):248-251. DOI:10.3969/j.issn.1673-5323.2006.05.003. |

**Appendix S4**. Summary of articles on costs of childhood cancer treatment among the Chinese population

| **Citation** | **Place** | **Study design** | **Study period** | **Sample size** |
| --- | --- | --- | --- | --- |
| Ren 2015 | Shanghai | Hospital-based cross-sectional | 2008-2010 | 161 patients |
| Chang 2015 | Lanzhou, Gansu Province | Hospital-based retrospective cohort | 2003-2012 | 260 patients |
| Wang 2019 | Zhengzhou, Henan Province | Hospital-based case-control | 2017-2018 | 79 patients |
| Huang 2019 | Tianjin | Hospital-based cross-sectional | 2015 | 78 patients |
| Zhao 2020 | Heyuan, Guangdong Province | Hospital-based cross-sectional | August 2017 to July 2019 | 45 patients |

**Detailed summary:**

There were five studies in the Chinese literature that estimated the care cost and economic burden associated with cancer treatment among patients actively receiving cancer therapies.^1-5^ As such estimation is crucial given cancer treatment is a strong driver of financial hardship during survivorship, we summarize these findings in Appendix S2.

In 2015, Ren and colleagues estimated direct and indirect costs of cancer treatment for 161 families of children with ALL -- who completed treatment courses at the Shanghai Children's Medical Center during 2008-2010 -- through a comprehensive review of the Center’s database supplemented with a survey to parents of the eligible patients.^2^ This study showed that the average cancer treatment costs were 222,800.67 CNY, comprising 51.67% direct medical expenses, 20.48% direct non-medical expenses (including expenses for transportation, lodging, and other costs directly associated with receipt of the cancer treatment), and 27.85% indirect costs (mainly productivity loss of families during cancer diagnosis and treatment). In a recent evaluation of 45 leukemia children treated for over a year in Heyuan, Guangdong Province, the average direct medical cost, direct non-medical cost, and indirect cost were 242,400 CNY (65.6%), 36,600 CNY (15.1%) and 90,500 CNY (24.49%), respectively.^1^ In another recent study of 78 children treated for acute leukemia in a tertiary blood disease specialty hospital in Tianjin in 2015, the average direct medical cost, direct non-medical cost, and indirect cost were 313,135.73 CNY (62.59%), 100,487.28 CNY (20.09%) and 86,666.17 CNY (17.32%), respectively.^3^

Costs of cancer treatment were found to be associated with a variety of factors. A study of 79 children treated for ALL in a tertiary Children’s Hospital in Zhengzhou, Henan Province in 2017 found that hospital infections more than doubled the inpatient expenses.^4^ An earlier study, including 260 leukemia children hospitalized in the First Affiliated Hospital of Lanzhou University, reported that hospitalization days per person, hospitalization cost per person, and average daily hospitalization cost all increased during 2003-2012, with annual percentage changes of 10.66%, 17.49%, and 6.15%, respectively.^5^

**Appendix References:**

1. Zhao G. *Study on the economic burden of leukemia children and the compensation effect of charity medical assistance -- a case study of Z project [M]*, Shandong Univeristy; 2020.

2. Ren Y, Ji Q, Zhang J, Zhang L, Li X. The Disease-related Burden of Families with Children with Acute Lymphoblastic Leukemia. *Chinese General Practice.* 2015(1):19-21.

3. Huang X, Zhang H, Yang M, et al. Investigation on Family Economic Burden and Objective Social Support of Children with Acute Leukemia. *Health Economics Research.* 2019;36(7):38-40.

4. Wang J, Zhang F. Study on the Effect of Nosocomial Infection on Hospitalization Expenses——Taking Children with Acute Lymphoblastic Leukemia as An Example. *Health Economics Research.* 2019;36(1):26-28.

5. Chang R, Chen Z, Xi Y, Wu S, Chen W, Li Z. Analysis of Direct Economic Burden in Hospitalized Leukemia Children from 2003 to 2012，Gansu Province. *Chinese General Practice.* 2015;18(5):569-572.
